# Prevalence and associated factors of intimate partner violence amongst women attending prevention of mother to child transmission services in Blantyre, Malawi

**DOI:** 10.4102/safp.v63i1.5271

**Published:** 2021-10-07

**Authors:** Lignet Chepuka, Chimwemwe Kwanjo-Banda, Ursula Kafulafula, Anthony Sefasi, Genesis Chorwe-Sungani

**Affiliations:** 1 Department of Adult Health, Faculty of Nursing Health Studies, Kamuzu College of Nursing, University of Malawi, Blantyre, Malawi; 2 Department of Midwifery, Faculty of Midwifery Studies, Kamuzu College of Nursing, University of Malawi, Blantyre, Malawi; 3 Department of Mental Health, Faculty of Community Health Studies, Kamuzu College of Nursing, University of Malawi, Blantyre, Malawi

**Keywords:** antenatal, depression, HIV-positive mothers, Intimate partner violence (IPV), perinatal

## Abstract

**Background:**

Intimate partner violence (IPV) during the perinatal period and when one is HIV-positive is a great concern because of the physical and mental impacts it has on health and on adherence to prevention of mother to child transmission (PMTCT) services. However, factors associated with IPV amongst perinatal women on PMTCT services are not adequately explored in Malawi. The aim of this study was to estimate the various types of IPV and the associated factors amongst HIV-positive pregnant and postnatal women in selected health centres in Blantyre district.

**Methods:**

In this cross-sectional study, we recruited 200 HIV-positive women from antenatal, postnatal and antiretroviral therapy (ART) clinics from four selected primary care facilities of Blantyre district. Data were collected between March and May 2018.

**Results:**

A total of 50% of the participants reported to have experienced either physical, psychological or sexual violence from their partner in the last 12 months. The multivariate logistic regression model showed that feelings about safety of the relationship and depression were the only factors that were consistently associated with IPV in the last 12 months (*p* = 0.001, Pseudo *R*^2^ = 0.20).

**Conclusion:**

The presence of depression and safety concerns amongst our study participants calls for serious prioritisation of psychological interventions and risk assessment in the management of HIV-positive perinatal mothers who report IPV cases.

## Introduction

Intimate partner violence (IPV) is of great concern not only from a human rights perspective but also from economic and health perspectives.^1^ Violence, especially from intimate partners, during pregnancy and when one is HIV-positive can lead to poor adherence to prevention of mother-to-child transmission (PMTCT) services.^2,3,4,5,6,7,8^ Perceived risk of existing violence may influence disclosure or partner notification by HIV-positive women.^9^ Intimate partner violence may be a barrier to behavioural modifications after knowing one’s sero-status, including use of barrier methods and breastfeeding practices, especially where there is poor couple communication and negotiation.^7^ Fear and experience of violence from partners shape women’s access and adherence to PMTCT, unequal gender relations and lack of support from partners are amongst other reasons women cite for dropping out of PMTCT or pre-antiretroviral therapy (ART) services.^10,11,12,13,14^

Estimates indicate that 90% – 95% participation in PMTCT strategies, including effective adherence to ART, can reduce the global infant HIV incidence below 5%.^15,16,17^ Although there has been high acceptability of PMTCT in Malawi, the attrition rate in PMTCT programme is highly unacceptable. Non-adherence to PMTCT programme occurs at all stages of the ante-, peri- and post-natal period^18^ and not all pregnant HIV-positive women receive ART treatment.^19^ A review of literature by United Nations International Children and Emergency Fund (UNICEF) revealed that PMTCT coverage is estimated at 65% in 21 African countries. This means that close to a third of HIV-positive women are uncovered and not all mothers on medication adhere to it.^20^

Although 95% of pregnant women attending antenatal care in Malawi were tested for HIV, only 45% of HIV-positive pregnant women and 34% of babies born to HIV-positive mothers received antiretroviral (ARV) prophylaxis.^21^ Tenthani and colleagues in 2014 found that 17% of all women who started ART under Option B+ initiates all pregnant or breastfeeding women on lifelong combination antiretroviral therapy (cART) regardless of their disease stage to prevent mother to child HIV transmission, were lost to follow-up 6 months after ART initiation at one of the 540 facilities that were followed from October 2011 to March 2012. The loss to follow-up was even higher (24%) in larger facilities with electronic medical record systems (EMRS). Women who started ART to prevent mother-to-child transmission (MTCT) during pregnancy were five times more likely never to return to the clinic after they initiated ART compared with those who started ART for their own health.^22^ Those who initiated ART whilst breastfeeding were twice as likely to not to return compared with those who started for their own health.^22^ The situation was similar for those who were initiated on ART on the day of testing. The risk of loss to follow-up was highest in facilities with large proportion of Option B+ patients.^22^

The effect of IPV on uptake and adherence to PMTCT services should not be undermined, especially in the case of Malawi, where IPV is highly prevalent ranging from 23% to 59%^23,24,25,26,27,28,29^ and normalised, as it is seen as an unavoidable tool in resolving conflicts (‘beating is the cure for marriage’),^27^ with ‘educational’ beating seen as a sign of love.^27^

Globally, literature shows that between 27.4% and 55.0% of women suffer from IPV in their lifetime.^1,30,31,32,33,34,35,36^ In Malawi, the 2015–2016 Malawi Demographic Health Survey (MDHS) shows that 42% of women experience IPV^1^ although a decrease from what Chasweka and colleagues^26^ at Nsanje district hospital revealed (59%) amongst women attending antenatal clinic but still much higher prevalence than many conditions routinely screened for during pregnancy.^37^

Intimate partner violence comes in different forms. The most commonly experienced IPV amongst women is emotional, also referred to as psychological violence.^1,31,33^ The other forms of IPV are physical and sexual violence. Although the prevalence of the three types of IPV amongst women in Malawi vary, the overall trend is consistent with emotional violence being the most commonly experienced (13% – 30%), physical (4% – 26%) and sexual violence (13% – 19%).^1,38^

Several factors contribute to increased risk of IPV amongst women. In a systematic review that was conducted in the United States (US), women who abused alcohol or substances were more likely to experience IPV than their counterparts.^30^ However, in a South African study, partner alcohol abuse was seen to pose women at risk of IPV.^39^ Women who experienced childhood abuse or witnessed their mothers being abused also reported IPV.^31,39^ Prevalence of IPV amongst women from cultures that practice male dominated relationships were higher than those from non-male dominated relationships.^38,40^ Studies from African settings^31,38,39,41,42^ including Malawi^38,43^ have shown that higher education has a protective factor against IPV amongst women. Furthermore, literature shows that those who enter into marriage at a tender age are likely to be abused by their intimate partners.^41,43,44^ However, factors associated with IPV amongst perinatal women on PMTCT services are not adequately explored in Malawi. Therefore, the aim of this study was to estimate the various types of IPV and the associated factors amongst HIV-positive pregnant and postnatal women in selected health centres in Blantyre district.

## Methods

### Study design

In this cross-sectional study, we recruited HIV-positive women from antenatal, postnatal and ART clinics from four selected primary care facilities of Blantyre district. Two of the facilities were located in an urban setting and the other two were rural. Data were collected between March 2018 and May 2018.

### Sample size considerations

An estimated 372 participants were needed to be recruited for the study. This sample size was calculated using a formula, which stipulates that:^2,45^

$$n=Z^{2}(p)(1-p)/\text{e},$$ [Eqn 1]

and is interpreted as follows: *n* = sample size, *Z* = value of a normally distributed variate, which for 95% confidence interval (CI) takes the value of 1.96 and *p* = estimated proportion of pregnant women experiencing IPV, which is 59% (based on a previous study on IPV amongst antenatal attendees). The antenatal study was used as a proxy because we could not access a study within the country that covered the perinatal period. We added description of severe or moderate abuse: The frequency of violence was considered severe if a person reported to have experienced any form of violence more than five times and moderate if violence occurred less than five times and there were no injuries sustained requiring medical attention. Where as *e* = desired precision or standard error, which was set at 0.05.

### Inclusion and exclusion criteria

All women who were pregnant or were within 6 weeks post-natal period were referred to the research team by nurses/midwives or data clerks at the clinics for screening if they met the inclusion criteria. Women were recruited into the study if they were aged 18 years and above, HIV-positive and consented to participate.

### Data collection and instruments

We collected data on the participants’ social demographics, relationship history, HIV and IPV risk factors and attitudes and perceptions about IPV based on a literature review of factors associated with IPV in HIV-positive perinatal women. The interviewers administered the questionnaires. The interviewer administered questionnaire was preferred because it could be administered to participants who were illiterate and allowed the interviewers to clarify questions for the participants. This resulted in fewer unanswered items and high response rate.

The abuse assessment screen (AAS) was used to screen for IPV.^46^ According to a literature of IPV screening tools, there is no gold standard for IPV screening as no single IPV screening tool has well-established psychometric properties. However, the AAS was preferred because it has been found to have a high Cronbach’s alpha amongst pregnant women. In a Greek study amongst pregnant women, the AAS had a Cronbach’s alpha of 0.806.^47^ In Spain, the AAS had good test-retest reliability, specificity and construct validity amongst pregnant women.^48^ The self-reporting questionnaire (SRQ) was used to screen for depression. The internal consistency of the SRQ amongst Malawian women was high (Cronbach’s alpha 0.825).^49^ The SRQ has 20 items to which participants answered yes if they experienced the symptoms or no if there were no symptoms.

### Data analysis

Data were entered into a data base created in International Business Machines Corporation (IBM) Statistical Package for Social Sciences (SPSS) version 19 and exported to Stata version 14.0 for analysis. Descriptive statistics were done to calculate frequency distributions for the categorical predictor variables and severity of different types of violence experienced. The outcome variable was experience of any form of IPV in the past 12 months. Univariate logistic regression analysis was done to investigate the independent association between the outcome variable (experience of IPV) and the predictor variables (participants’ social demographics, relationship history, HIV and IPV risk factors and attitudes and perceptions about IPV). Chi-square (or Fisher’s exact) test were used for testing association between the binary outcome of IPV experience and the categorical predictor variables. Factors that showed association with experience of IPV at alpha 0.1 or less were included in the multivariate logistic regression model adjusting for participants age and education status as possible confounders. The results are presented in Table 5.

## Findings

A total of 410 pregnant and postnatal mothers were screened for recruitment from all the four facilities. Only 207 of them met the inclusion criteria, of which 200 consented to participate in the study, giving a response rate of 96.6%. Over half (52.5%) of the participants were pregnant and 47.5% were post-natal mothers. About half (51%) were below the age of 28 years. A total of 117 (58.5%) participants attained primary school education, 178 (89%) were Christians and 22 (11%) were Muslims. Most of them (63.5%) were unemployed and 57% of the participants had at least one child. Table 1 details the demographic characteristics of the 200 participants.

**TABLE 1:** Demographic characteristics of the participants.

Variable	Frequency	
	*n*	%
**Age**		
≤ 28	102	51.00
29 >	98	49.00
**Level of education**		
None	7	3.50
Primary	117	58.50
Secondary	71	35.50
Post-secondary	4	2.00
Not indicated	1	0.50
**Religion**		
Christian	178	89.00
Muslim	22	11.00
**Occupation**		
Student	3	1.50
Unemployed	127	63.50
Paid employees	22	11.00
Self-employed	48	24.00
**Number of children**		
No children	20	10.00
1–4 children	114	57.00
5 and above	66	33.00
**Age of children**		
0–18	187	93.50
Above 18	13	6.50
**Currently pregnant**		
Yes	106	53.00
No	94	47.00
**Gestational age**		
First trimester	14	13.21
Second trimester	23	21.70
Third trimester	35	33.02
Not known	34	32.08

A total of 189 (94.5%) of the participants reported that they had an intimate partner. About half of them (50.5%) had been in the relationship for less than 5 years. A total of 74.0% were in a monogamous marriage, 8% were in a polygamous marriage, 10.0% were cohabiting, 4.7% were in a stable relationship (a recognised boyfriend to the family) but not staying together and 1% were in a causal relationship (non-committal sexual relationship) whilst 1.5% were divorced. A total of 100 (50.0%) reported that there was no tension in their relationship whilst the other 50.0% reported that there was some tension or a lot of tension in their relationship. Only 33.5% of the participants reported feeling safe in their current relationship. Over 60.0% of the partners attended secondary school education. A total of 80.0% of them were Christians and 93.5% were engaged in some form of employment. Of these, 44.0% were on paid employment. Table 2 shows the characteristics of the partners.

**TABLE 2:** Characteristics of the partners to the participants.

Variable	Frequency	
	*n*	%
**Partner education**		
None	8	4.0
Primary	40	20.0
Secondary	123	61.5
Post-secondary	11	5.5
**Partner religion**		
Christian	160	80.0
Muslim	22	11.0
Other	18	9.0
**Partner occupation**		
Unemployed	13	6.5
Paid employees	88	44.0
Self-employed	85	42.5
Other	14	7.0

More than 87% of the participants did not find IPV justifiable if a woman went out without letting her husband know, failed to care for the children, answered back to her husband during an argument, refused sex or burnt food. Table 3 shows the proportion of participants who found some situations justifiable for a man to beat a woman.

**TABLE 3:** Attitudes towards intimate partner violence.

Is a man justified to beat a woman for the following reasons	Frequency	
	*n*	%
**Going out without letting husband know**		
Yes	25	12.5
No	175	87.5
**Failing to care for the children**		
Yes	22	11.0
No	178	89.0
**Answering back during an argument**		
Yes	20	10.0
No	180	90.0
**Refusing sex**		
Yes	9	4.5
No	191	95.5
**Burning food**		
Yes	7	3.55
No	190	96.45

### Experience of violence

Half (50.0%) of the participants reported to have ever experienced some form of IPV in their present or past relationship. A total of 14% reported to have ever experienced physical violence whilst pregnant with the current or previous pregnancy. The frequency of violence was considered severe if a person reported to have experienced any form of violence more than five times and moderate if violence occurred less than five times and there were no injuries sustained requiring medical attention. In the past 12 months, 19.0% of the participants experienced physical violence, 20.0% experienced sexual violence and 31.5% experienced emotional or psychological violence. In the past 6 months, 15.6% experienced physical violence, 16.6% experienced sexual violence and 26.6% experienced emotional or psychological violence. In the past three months, 12.6% experienced physical violence, 16.0% experienced sexual violence and 21.6% experienced emotional or psychological violence. Table 4 shows the frequency and severity of experiencing physical, emotional and sexual violence.

**TABLE 4:** Frequency of experiencing severity of physical, emotional and sexual violence.

Variable	Frequency	
	*n*	%
**Experience of physical abuse past year**		
None	161	82.14
Moderate	26	13.27
Severe	9	4.59
**Experience of sexual violence past year**		
None	164	82.00
Moderate	10	5.00
Severe	26	13.00
**Experience of emotional violence past year**		
None	135	68.53
Moderate	40	20.00
Severe	22	11.00
**Experience of physical violence past 6 months**		
None	168	84.42
Moderate	22	11.06
Severe	9	4.52
**Experience of sexual violence past 6 months**		
None	166	83.84
Moderate	14	7.07
Severe	18	9.09
**Experience of emotional violence past 6 months**		
None	146	73.37
Moderate	32	16.08
Severe	21	10.55
**Experience of physical violence past 3 months**		
None	174	87.44
Moderate	15	7.54
Severe	10	5.03
**Experience of sexual violence past 3 months**		
None	167	83.92
Moderate	15	7.54
Severe	17	8.54
**Experience of emotional violence past 3 months**		
None	156	78.39
Moderate	23	11.56
Severe	20	10.05

Overall, 87 (43.7%) of the participants had experienced physical, emotional or sexual violence in the past 1 year. In the univariate logistic regression analysis, factors that were independently associated (*p* < 0.05) with experience of violence in the past 12 months were pregnancy, duration of relationship, nature of relationship, partner religion, feeling about safety of relationship, willingness to participate in IPV prevention groups and depression.

After adjusting for age and educational level of the participants as possible confounders, the multivariate logistic regression model showed that feelings about safety of the relationship and depression were the only factors that were associated with IPV in the last 12 months (*p* = 0.001, Pseudo *R*^2^ = 0.20). Table 5 shows the results of the multivariate logistic regression analysis.

**TABLE 5:** Multivariate logistic regression model.

Variable	Odds ratio	95% CI	*p*-value
**Age**			
≤ 28	Ref.	-	-
29 >	1.764	0.811–3.838	0.152
**Education**			
Primary or less	Ref.	-	-
Secondary and above	1.167	0.562–2.422	0.679
**Pregnant**			
Yes	Ref.	-	-
No	1.333	0.623–2.852	0.459
**Relationship duration**			
< 1 year	Ref.	-	-
1–2 years	0.245	0.056–1.080	0.063
3–5 years	0.7182	0.169–3.048	0.654
> 5 years	0.491	0.130–1.850	0.293
Not applicable	0.521	0.027–10.156	0.667
**Nature of relationship**			
Married monogamous	Ref.	-	-
Married polygamous	0.918	0.056–1.080	0.897
Steady partner not living together	0.514	0.169–3.048	0.418
Steady partner living together	0.391	0.130–1.850	0.222
Causal relationship	1.757	0.027–10.156	0.750
**Number of sex partners**			
One	Ref.	-	-
Did not disclose	0.752	0.201–2.809	0.672
Three	0.138	0.012–1.614	0.115
**Partner religion**			
Christian: Catholic	Ref.	-	-
Christian: Protestant	0.721	0.314–1.661	0.444
Muslim	0.819	0.237–2.832	0.752
Other	0.992	0.153–6.457	0.994
**Feels safe in relationship**			
Yes	Ref.	-	-
No	2.985	1.334–6.682	0.008*
**Opinion about IPV enquires during ANC visits**			
Yes	Ref.	-	-
No	1.842	0.292–11.625	0.516
**Willingness to join any group**			
Yes	Ref.	-	-
No	2.854	0.629–12.946	0.174
**Depressed**			
Yes	Ref.	-	-
No	5.826	1.908–17.789	0.002*

ANC, antenatal clinic; CI, confidence interval; IPV, intimate partner violence; Ref., reference.

* , *p* < 0.01.

## Discussion

In our sample of 200 HIV-positive women, 50% reported to have experienced various forms of IPV in the last 12 months. This finding is comparable with findings from previous studies around the globe where the prevalence of IPV ranged from 27.4% to 55.0%,^1,29,30,31,32,33,34,36^ but slightly lower than those obtained amongst antenatal attendees from Nsanje district hospital in Malawi, which reported prevalence of 59.0%.^26^ In addition, our participants reported high prevalence of sexual (18%) than physical IPV (17.86%), which is a departure from previous studies, which rated physical IPV higher than sexual IPV.^1,38,43^ Although the difference observed here is not statistically significant, a more plausible explanation would be that more women are now becoming aware of their rights and are removing the veil of silence that shrouded the issues of sexual violence within the intimate relationship.

Our study also revealed that the most common form of violence women experienced across the different reporting cut-off points (from 1 year, 6 months and 3 months) was emotional or psychological in nature (31%). The higher rates of psychological violence is of major concern considering that at present, it is well-documented that IPV can cause extensive mental health consequences amongst its victims such as symptoms of post-traumatic stress disorder (PTSD) along with other comorbid symptoms such as depression, anxiety, suicidality, substance^50^ abuse and sleep disturbances and can interrupt uptake and adherence to PMTCT services.

When we did univariate logistic regression analysis, we found the factors that were independently associated (*p* < 0.05) with experience of violence in the past 12 months were pregnancy, duration of relationship, nature of relationship, partner religion, feeling about safety of relationship, willingness to join IPV prevention groups and depression. However, woman’s education and that of the partner were not independently associated with IPV as was the case with previous studies conducted in other African countries^31,38,39,41,42,43^ including Malawi^38,43^ where higher education was protective against IPV.

Considering the high levels of psychological violence in this study, it was not surprising that after adjusting for age and educational level of the participants as possible confounders, the multivariate logistic regression model showed that feelings about safety of the relationship and depression were the only factors that were associated with IPV in the last 12 months (*p* = 0.01, Pseudo *R*^2^ = 0.20). Women who reported that they don’t feel safe in their relationship and those found to be depressed using the SRQ were more likely to have experienced any form of violence in the last 12 months. Our study is not the first to find a consistent relationship between IPV and depression as previous studies from Malawi^47,48,49^ and elsewhere^50^ have made similar observations amongst pregnant women, and more so, amongst those experiencing IPV^51^ and are HIV-positive.^52,53^ However, our study’s finding on the relationship between safety in a relationship and IPV is in contrast to an earlier study in primary care, which found that many women who reported experiencing violence in their relationship did not report feeling unsafe at home.^54^

The difference between our study and the other studies^54^ is that we recruited women with the triple burden for depression (perinatal, IPV experience and HIV-positive). Unfortunately, tools for screening depression have been validated in the country^47,53,56^ but mental health services for such women are non-existent as perinatal health services in the country are biased towards provision of physical health^55,57^ and routine screening for maternal depression is not done.

In addition, our study’s findings underscore the importance of conducting risk assessment for clients reporting any form of violence. A recent study supported the validity of both structured risk assessment tools and victim perceptions as predictors of risk for repeated IPV.^46^ Combining structured risk assessments and victim risk assessments leads to better predictions of repeat violence than when performed separately, suggesting that the two forms of assessment provide unique and complementary information. However, these tools have not been tried in our settings and there might be a need for psychometrically testing these to ensure their sensitivity.

## Conclusion

Our research demonstrates that depression and safety issues are major problems that perinatal mothers who are HIV-positive encounter and also points to what can be learned about the changing dynamics in the variables that really matter with regard to our understanding of IPV. However, one limitation of our study is that it was a cross-sectional study, as such we cannot confirm the direction of influence between IPV and the associated factors. Nonetheless, the presence of depression and safety concerns amongst our study participants calls for serious prioritisation of psychological interventions and risk assessment in the management of HIV-positive perinatal mothers who report IPV cases. There might be a need to also validate the risk assessment tools and IPV screening tools within these settings to ensure that they are culturally responsive and include a question on safety as a proxy for screening violence in relationships. In addition, the results also suggested that participants who had experienced violence are more likely to express interest to join IPV prevention support groups. Healthcare providers will have to be trained to gain knowledge and skills on how to address IPV situations as they arise in the context of their duty.
